# Screening and construction of nanobodies against human CD93 using phage libraries and study of their antiangiogenic effects

**DOI:** 10.3389/fbioe.2024.1372245

**Published:** 2024-05-01

**Authors:** Hui Miao, Yiling Wu, Hao Ouyang, Peiwen Zhang, Wenyun Zheng, Xingyuan Ma

**Affiliations:** ^1^ State Key Laboratory of Bioreactor Engineering, East China University of Science and Technology, Shanghai, China; ^2^ Department of Hepatology, Yueyang Hospital of Integrated Traditional Chinese and Western Medicine, Shanghai University of Traditional Chinese Medicine, Shanghai, China; ^3^ Shanghai Key Laboratory of New Drug Design, School of Pharmacy, East China University of Science and Technology, Shanghai, China

**Keywords:** CD93, nanobody, phage display, angiogenesis, vascular permeability

## Abstract

**Background:**

Cluster of Differentiation 93 (CD93) plays an important role in angiogenesis and is considered an important target for inhibiting tumor angiogenesis, but there are currently no therapeutic antibodies against CD93 in the clinic. Thus, we describe the screening of novel nanobodies (Nbs) targeting human CD93 from a phage library of shark-derived Nbs.

**Methods:**

Screening and enrichment of phage libraries by enzyme-linked immunosorbent assay (ELISA). Anti-CD93 Nbs were purified by expression in *E. coli*. The binding affinity of anti-CD93 Nbs NC81/NC89 for CD93 was examined by flow cytometry (FC) and ELISA. The thermal stability of NC81/NC89 was examined by ELISA and CD spectroscopy. Afterward, the anti-angiogenic ability of NC81/NC89 was examined by MTT, wound healing assay, and tube formation assay. The expression level of VE-cadherin (VE-Ca) and CD93 was detected by Western Blot (WB). The binding sites and binding forms of NC81/NC89 to CD93 were analyzed by molecular docking.

**Results:**

The anti-CD93 Nbs were screened in a phage library, expressed in *E. coli*, and purified to >95% purity. The results of FC and ELISA showed that NC81/NC89 have binding ability to human umbilical vein endothelial cells (HUVECs). The results of ELISA and CD spectroscopy showed that NC81/NC89 retained the ability to bind CD93 at 80°C and that the secondary structure remained stable. *In vitro*, the results showed that NC81 and NC89 significantly inhibited the proliferation and migration of human umbilical vein endothelial cells (HUVECs) as well as tube formation on Matrigel. Western Blot showed that NC81 and NC89 also inhibited the expression of VE-Ca thereby increasing vascular permeability. It was found during molecular docking that the CDR regions of NC81 and NC89 could be attached to CD93 by strong hydrogen bonds and salt bridges, and the binding sites were different.

**Conclusion:**

We have successfully isolated NC81 and NC89, which bind CD93, and both Nbs significantly inhibit angiogenesis and increase vascular permeability. These results suggest that NC81 and NC89 have potential clinical applications in angiogenesis-related therapies.

## Introduction

The cluster of Differentiation 93 (CD93) is a C-type lectin transmembrane receptor that plays an important role in embryonic development, immune cell function, inflammation, cancer, and angiogenesis (Greenlee-Wacker et al., 2012; Langenkamp et al., 2015; Lugano et al., 2018; Nativel et al., 2019; Liang et al., 2020; Sun et al., 2021). CD93 was originally thought to be a receptor for Clq but is now thought to be involved in intercellular adhesion and clearance of apoptotic cells (Carmeliet, 2003; Greenlee et al., 2008). The intracellular cytoplasmic tail of this protein contains two highly conserved structural domains that may be involved in the function of CD93. In angiogenesis, CD93 can promote the migration and growth of vascular endothelial cells and smooth muscle cells by binding to collagen in peripheral tissues, which in turn is involved in the formation of new blood vessels (Zhang et al., 2005). A recent study has shown that CD93 is downregulated in the context of VEGF inhibition and is a potential target for mediating vascular normalization (Lugano et al., 2023). Thus, CD93 has become a hot topic of research as an important molecule in the regulation of angiogenesis and related diseases.

Antibodies are usually obtained by immunizing the animal with the antigen and obtaining specific antibodies from peripheral blood. This method is costly and time-consuming, and antibodies can only be obtained against one antigen. Another method is to collect peripheral blood from unimmunized camels and extract the total RNA to construct a natural library that can be used directly for antibody screening, but the antibodies obtained in this way are usually not very immunospecific (Hoogenboom, 2005). To obtain highly specific antibodies more easily and quickly, synthetic libraries based on genetic engineering techniques and bioinformatic design of phage display have been designed and are now increasingly used for screening antibodies to specific antigens and for antibody *in vitro* affinity maturation (Mahdavi et al., 2022).

Most of the antibodies available on the market today are monoclonal antibodies (mAbs), and with the widespread use of mAbs in the clinic (Lu et al., 2020; Wang et al., 2022), their limitations have come to light, such as their large molecular weight, which leads to poor tissue penetration, as well as their long lead time and high cost of preparation. In 1993, Hamers discovered a heavy chain antibody from camelids that lacked a light chain, of which the heavy chain variable region VHH was called nanobody (Nb) (Hamers-Casterman et al., 1993). In 1995, GreeNberg et al. identified another VNAR in sharks that was similar to the camelid heavy chain antibody (Greenberg et al., 1995). Compared to mAbs, Nbs have many advantages due to their small molecular weight, such as high tissue penetration, high stability, ease of purification, rapid production, and low cost. Nb is now widely used in diagnostics, disease detection, and cancer treatment. The use of Nb can increase the cost and efficiency of experiments.

## Materials and methods

### Chemicals and antibodies

TG1 cells were purchased from Lucigen (Wisconsin, America) *E. coli* DH5α, and E. coli BL21 (DE3) were purchased from Weidibio (Shanghai, China). The restriction enzymes, T4 DNA ligase, and PrimeSTAR^®^ HS DNA Polymerase were purchased from Takara (Tokyo, Japan). HRP-conjugated anti-HA tag monoclonal antibody was purchased from Sino Biological (Beijing, China). Fetal bovine serum (FBS), DMEM, ECM, and penicillin-streptomycin (10,000 U/mL penicillin and 10 mg/mL streptomycin) were obtained from Thermo Fisher Scientific (Waltham, USA). Antibodies for VE-cadherin (VE-Ca), and β-actin were all purchased from Cell Signaling Technology (Danvers, MA). Anti-CD93 antibody was bought from Santa Cruz (Santa Cruz, CA). Anti-His antibody was bought from Proteintech (Chicago, USA). 488-conjugated Goat Anti-Mouse IgG (H + L) antibody was purchased from ABclonal (Wuhan, China). Other reagents unless indicated were obtained from Sigma Chemical Co. (St. Louis, MO).

### Cell culture

Human embryonic kidney cells (HEK293) preserved in our lab were cultured in DMEM (high glucose), supplemented with 10% (v/v) fetal bovine serum (FBS), 100 U/mL penicillin, and 100 mg/mL streptomycin. Human umbilical vein endothelial cells (HUVECs) purchased from ScienCell (Carlsbad, CA) were cultured in ECM supplemented with 5% (v/v) FBS and 1% (v/v) ECGS, 100 U/mL penicillin and 100 mg/mL streptomycin. Cells were incubated at 37 °C in a humidified atmosphere (5% CO2).

### Enrichment of anti-CD93 VHH phages through panning

The phage library was expanded and then infected with M13 helper phage (10^12^ CFU/mL) for 1 h. The samples were centrifuged at 8,000 rpm for 5 min, and the bacteria were resuspended in 200 mL of 2×TY medium (containing 100 μg/mL Amp and 50 μg/mL Kana) and incubated at 37°C overnight. The phage display library was recovered from the supernatant by precipitation with PEG/NaCl (20% PEG and 2.5 M NaCl). The ELISA plates were coated with CD93 (200 nM for the first and second rounds, 100 nM for the third and fourth rounds of biopanning) and incubated overnight at 4°C. After washing, 5% BSA solution (0.1% TBST dissolved) was added and incubated for 1 h at room temperature. After washing, prepared phage libraries were added to the wells and incubated for 2 h. The samples were incubated with a glycine-hydrochloric acid solution (20 mM Tris-HCl and 1% BSA, pH = 2.2) for 10 min at room temperature, collected in EP tubes, and immediately neutralized with Tris-HCl buffer to pH = 7.4. After that, infect the eluted phage with TG1. Phage amplification and titer assays were performed to prepare for the next round of panning.

### Phage ELISA and clone sequencing

Monoclonal phage ELISA was selected to determine the positive anti-CD93 Nbs clones. The ELISA plates were coated with 100 nM CD93 and BSA incubated overnight at 4°C. After washing, 5% BSA solution (0.1% TBST dissolved) was added and incubated for 1 h at room temperature. After washing, different phage monoclonals were added to the wells and incubated for 2 h. After washing, HRP-conjugated anti-M13 antibody was added and incubated for 1 h, after discarding the secondary antibody and washing, TMB solution was added to each well and incubated at 37°C for 30 min. At this time, the solution turned blue. After incubation, the reaction was terminated by adding 1M HCl, and the 450 nm absorbance value was measured in an enzyme calibrator. The obtained positive clones were cultured at 37°C for 4 h and sent for sequencing (Qing Ke, China). Protein sequences were translated by Expasy (Duvaud et al., 2021).

### Expression and purification of NC81 and NC89

The amplified gene product (Nde I-HA-Nb-Xho I) was purified, cleaved with Nde I and Xho I enzymes, and ligated into the pET-24a (+) plasmid. Recombinant vectors were transformed into *E. coli* BL21 (DE3). Cells were induced with 0.75 mM IPTG at 30°C for 10 h. The collected cells were crushed with the high-pressure homogenizer. Purification with Ni-columns, followed by dialysis desalting of the protein solution is essential. Finally, the protein purity was analyzed by SDS-PAGE electrophoresis.

### MTT assay for cell viability and proliferative capacity

HUVECs or HEK293 cells (4 × 10^3^) were seeded into 96-well plates and incubated for 24 h. Then, different concentrations of Nbs (0, 0.0001, 0.01, 0.1, 1, 10, 100, 1,000, and 10,000 nM) were added. After incubating at 37°C for 24 and 48 h, MTT solution (final concentration 0.5 mg/mL) was added and left for 4 h to form blue-purple methanate crystals. Then a triplet (10% SDS-5% isobutanol-0.01 M HCl) was added and left overnight to fully dissolve the crystals. The absorbance values at 570 nm and 630 nm (OD) were read. The percentage of absolute absorbance values between the drug administration group and the control group was determined as the cell viability.

### Affinity analysis

The affinity of anti-CD93 Nb was determined by indirect-ELISA (Zhang et al., 2016). The ELISA plates were coated with 5 μg/mL CD93 and BSA and incubated overnight at 4°C. After washing, 5% skimmed milk solution (0.1% TBST dissolved) was added and incubated for 1 h at room temperature. After washing, different concentrations of Nb were added to the wells and incubated for 2 h. After washing, HRP-conjugated anti-HA antibody was added and incubated for 1 h, After discarding the secondary antibody and washing, TMB solution was added to each well and incubated at 37°C for 30 min. At this time, the solution turns blue. After incubation, the reaction was terminated by adding 1M HCl, and the 450 nm absorbance value was measured in an enzyme calibrator.

### Flow cytometry assay

HUVECs or HEK293 (4 × 10^5^) cells per sample were suspended in PBS containing 5% BSA and then incubated with/without 10 μM (10 μM, which had reached the plateau phase, was selected for cell binding according to the ELISA assay.) NC81/NC89 at 37°C for 1 h. Resuspend the cells collected by centrifugation. The cells were washed and incubated with anti-His antibody for 1 h. After washing, the cells were incubated with a fluorescent secondary antibody for 1 h. Finally, the cells were washed and the binding assay was performed with a FACS flow cytometer.

### Wound healing assay

Ibidi inserts were fixed in a 24-well plate, and 5 × 10^3^ HUVECs were placed into a 24-well plate and then starved of serum-free ECM for 12 h. When the cells were grown in the chamber to 70% confluence, the insert was removed vertically and washed 1-2 times with DPBS. Three random photographs were then taken immediately under the microscope noted as 0 h. Complete medium containing different concentrations of Nbs (0, 0.1, 1, 5, and 10 μM) were added to each well. 12 h later, three random photographs of the scratch location of each well were taken with an OlYMPUS inverted microscope. The wound migrated distances were measured with ImageJ.

### Tube formation assay

Change the complete medium to serum-free medium 1 day in advance and incubate for 12 h. Matrigel (BD Biosciences) was thawed on ice for 24 h, then 50 μL Matrigel per well was coated into a 96-well tissue culture plate and incubated at 37 °C for 0.5 h to solidify. 1 × 10^4^ HUVECs were seeded into the 96-well plate and added with 200 μL ECM supplemented with 1% (v/v) FBS, 1% (v/v) ECGS and different concentrations of Nbs (0, 1 and 10 μM). Incubated at 37°C for 3–12 h, and the formation of endothelial tubes was photographed with an inverted OlYMPUS microscope at ×100 magnification. The endothelial tubes were counted with ImageJ.

### Western-blot analysis

Samples were separated by SDS-PAGE gel electrophoresis and electrophoretically transferred onto PVDF membranes, and then the membranes were probed with the appropriate combination of β-actin (1:1,000), CD93 (1:200), VE-Ca (1:1,000) and horseradish peroxidase-conjugated secondary antibodies. Proteins in the membranes were visualized by enhanced chemiluminescence kits. The protein bands were quantified by the average ratios of integral optic density following normalization to the expression of internal control β-actin, CD93, or VE-Ca, and the results were further normalized to control.

### Molecular docking

The protein sequences were submitted to the I-TASSER webserver for homology modeling using default settings. According to the evaluation results, the highest-score model was adopted for molecular docking. HADDOCK software was used to perform molecular docking for NC81/NC89 and CD93 (van Zundert et al., 2016; Honorato et al., 2021). The solvent-exposed residues of CD93 were analyzed by WHATIF tools and the CDR residues of NC81/NC89 were used. Based on the evaluation of the feedback docking clusters, the docking cluster with the smallest Z-score was the best, and the amino acid residues of CD93 interacting with NC81/NC89 were further analyzed by PDBePISA.

### Statistical analysis

Data are expressed as the mean ± standard error of the mean (SEM). Differences between groups were assessed by non-parametric one-way analysis of variance (ANOVA) followed by the least significant difference (LSD) *post hoc* test when ANOVA found a significance value of F and no variance inhomogeneity. Otherwise, Mann-Whitney U non-parametric ANOVA was performed. *p* < 0.05 is considered to be statistically significant.

## Results

### Panning and identification of anti-CD93 Nbs

**FIGURE 1:**
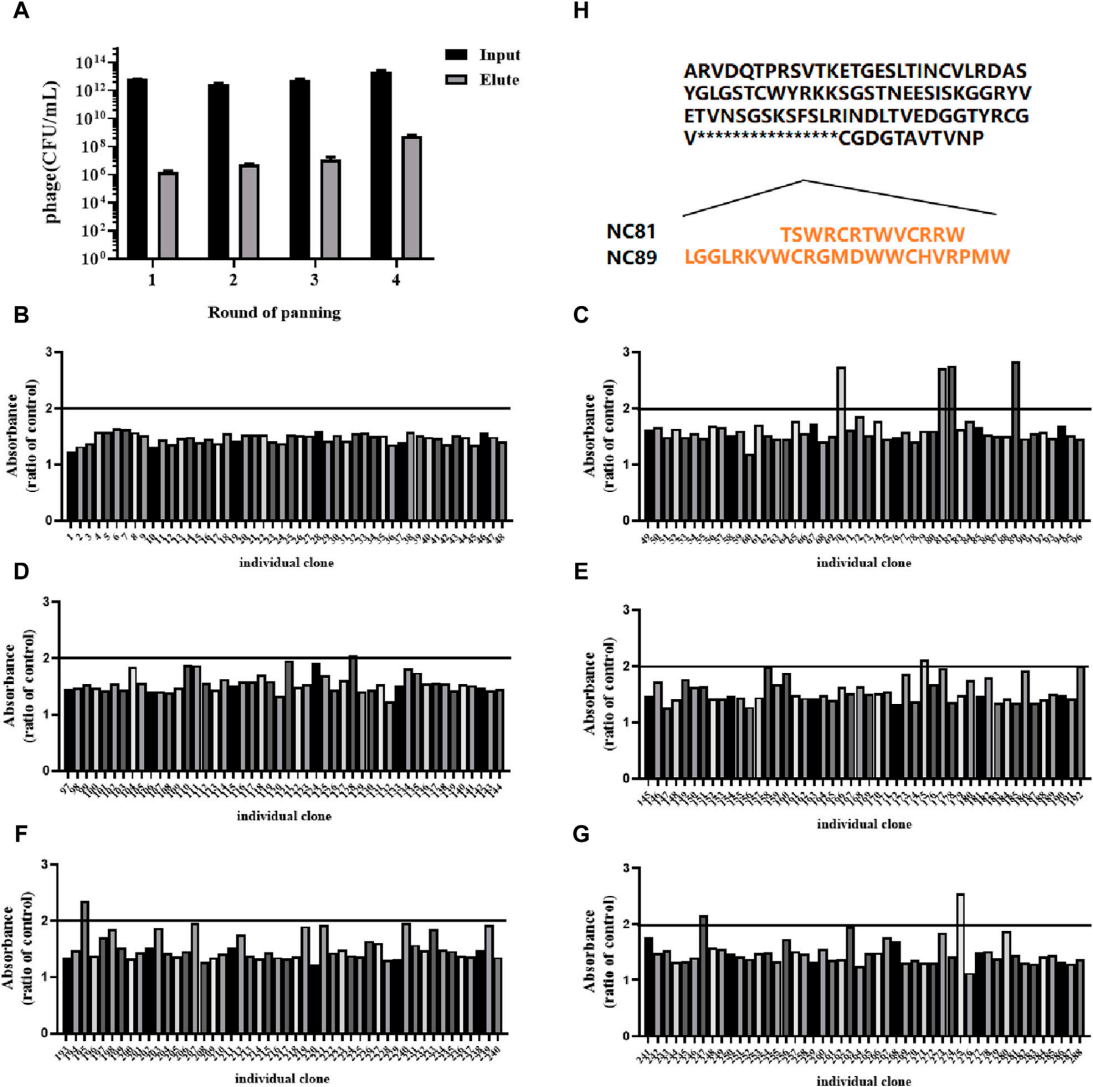
Anti-CD93 Nbs screening and phage ELISA. **(A)** The antigen-coated was CD93, and the negative control was coated with BSA. OD450 nm values were measured three times. **(B–G)** Analysis of CD93-binding phage by phage ELISA. The coated antigen was CD93, and the negative control was skimmed milk powder. The ratio of absorbance was calculated to obtain the positive monoclonal **(H)** Sequence of different positive monoclonals Nbs. Data are expressed as mean ± SEM.

CD93-specific Nbs were screened by solid-phase screening (Zhang et al., 2016), and phage titer assays showed an approximately 100-fold increase in antigen-bound clones in the fourth round compared to the first round (Table 1), indicating a large enrichment of clones bound to CD93 in the fourth round (Figure 1A). From the fourth enrichment, 288 monoclonal phage Nbs were selected and better monoclonal phages of anti-CD93 were selected by monoclonal phage ELISA (Figures 1B–G). As T/N ratios >2.0 were considered positive clones (Yan et al., 2014), a total of nine clones had ratios higher than 2 (Figures 1B–G). All positive clones were sent to the company for sequencing, and the only difference between the 2 Nbs (NC81 and NC89) was the different CDR3 sequences and lengths (Figure 1H).

**TABLE 1:** Enrichment Factors During Panning. Ransducing units (tu) before and after panning rounds were determined using titration of transduced colonies. Recovery rate, i.e., outputs. Input in the same round, and enrichment factor, i.e., the factor by which the ratio of rescued phages differed from round n-1 to round were calculated.

Round	Input (CFU/mL)	Elute (CFU/mL)	Recovery rate
1	6.4 × 10 ^12^	1.5 × 10^6^	2.3 × 10–^7^
2	2.9 × 10 ^12^	5.4 × 10 ^6^	1.9 × 10–^6^
3	5.9 × 10 ^12^	1.1 × 10 ^7^	1.86 × 10–^6^
4	2.1 × 10 ^13^	6.1 × 10 ^8^	2.9 × 10–^5^

### Anti-CD93 Nbs expression and purification

**FIGURE 2:**
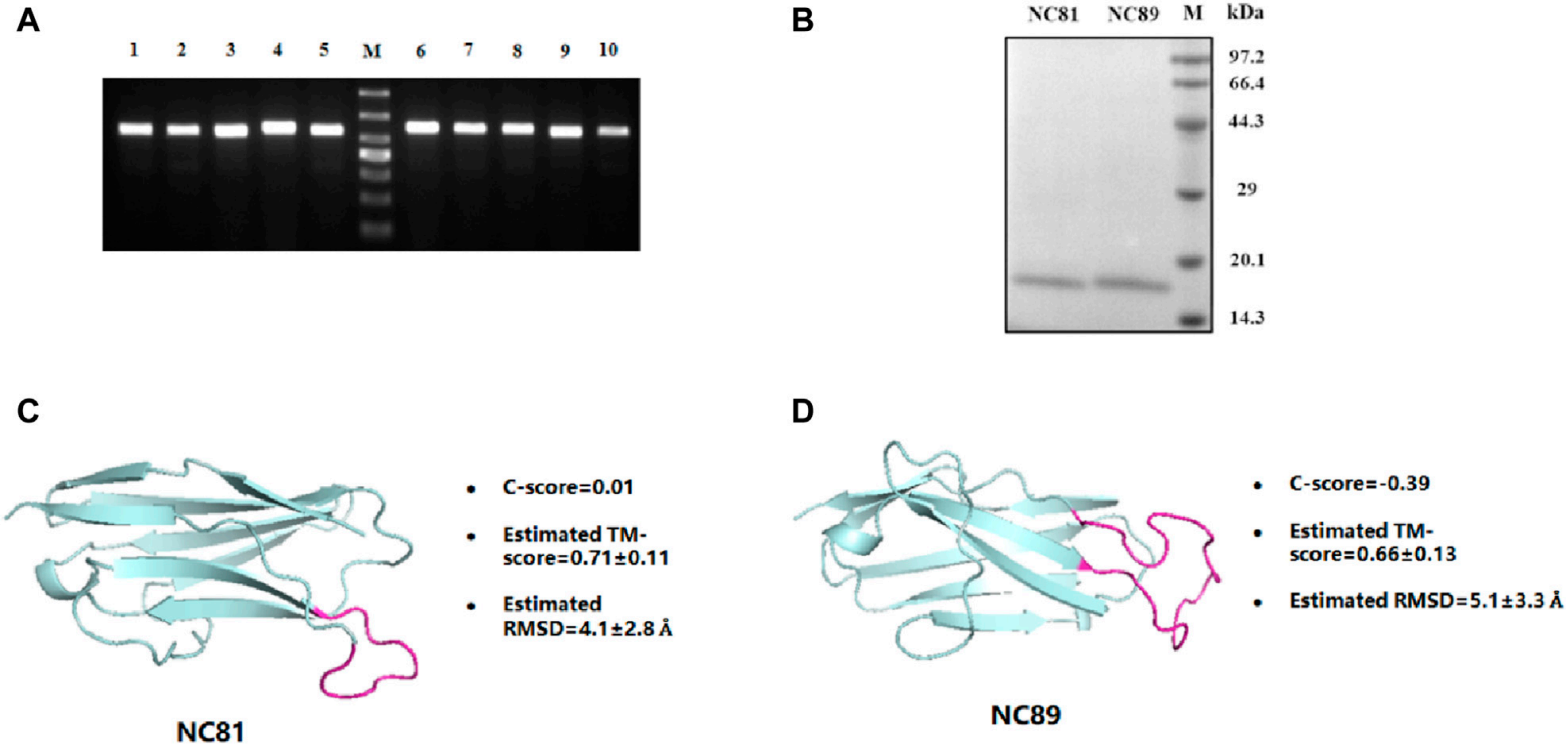
Anti-CD93 Nbs construction and expression purification. **(A)** PCR analysis of phagemid insert size. M, molecular weight marker. From top to bottom, 1,000 bp, 700 bp, 500 bp, 400 bp, 300 bp, 200 bp, 100 bp; lane 1-5, NC81; lane 6-10, NC89. There was a predicted 540/564 bp band in each lane. **(B)** SDS-PAGE analysis of purified anti-CD93 Nbs NC81 and NC89. Lane M: protein marker. **(C)** The model structures of anti-CD93 Nbs NC81 were obtained by I-Tasser. **(D)** The model structures of anti-CD93 Nbs NC89 were obtained by I-Tasser. Protein structures are shown in PYMOL.

The Nb gene was correctly inserted into the PET24a (+) vector by polymerase chain reaction (PCR) (Figure 2A). The recombinant protein was expressed in *E. coli*. BL21 (DE3) after induction by 1 mM IPTG at 30°C for 10 h. After purification by Ni-NTA affinity chromatography, the protein purity was greater than 95% by SDS-PAGE analysis (Figure 2B). In addition, the protein concentration of Nbs was determined, and the yield of Nbs ranged from 2.35 to 8.6 mg/L. The sequence of NC81/NC89 was simulated with I-TASSER and the corresponding tertiary structure of NC81/NC89 was obtained. (Yang and Zhang, 2015). The C-score in the simulation structure is usually between −5 and 2, with the highest C-score value indicating a high confidence level. In addition, a TM-score >0.5 usually indicates the model with the correct topology. As shown in Figures 2C, D, the optimal NC81 model had a C score of −0.81, RMSD of 6.0 ± 3.7 Å, and estimated TM of 0.61 ± 0.14, and the best model of NC89 had a C-score of −0.39, RMSD of 5.1 ± 3.3 Å and estimated TM of 0.66 ± 0.13. These evaluations showed that the obtained NC81/NC89 structural model was highly credible and that the CDR3 regions of both NC81/NC89 were exposed, favoring the binding of CD93 (Figures 2C, D).

### Affinity assay of anti-CD93 Nbs

**FIGURE 3:**
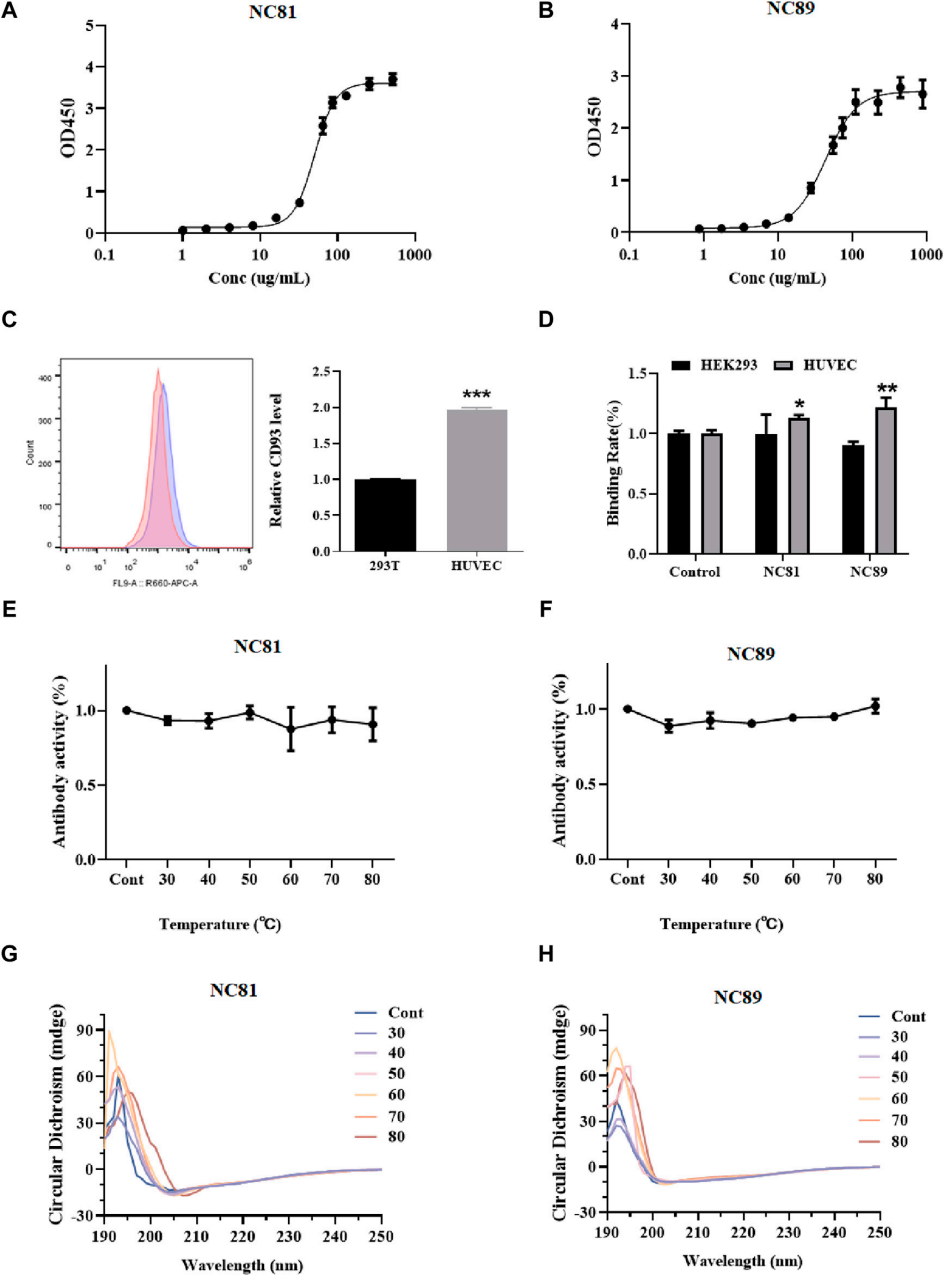
Anti-CD93 Nbs affinity and stability assays. **(A)** NC81 affinity assay by ELISA. The logistic curve fitting was analyzed by GraphPad (n = 3). **(B)** NC89 affinity assay by ELISA. The logistic curve fitting was analyzed by GraphPad (n = 3). **(C)** CD93 expression levels on the surface of HUVECs and HEK293 (n = 3). **(D)** Binding capacity of anti-CD93 Nbs to HUVECs and HEK293 (n = 3). **(E)** The binding capacity of NC81 to CD93 under different temperature treatments was determined by ELISA (n = 3). **(F)** The binding capacity of NC89 to CD93 under different temperature treatments was determined by ELISA (n = 3). **(G)** Secondary structure of NC81 under different temperature treatments. **(H)** Secondary structure of NC89 under different temperature treatments. Data are expressed as mean ± SEM.

To evaluate the affinity of anti-CD93 Nbs, indirect ELISA was performed to calculate the value of the equilibrium association constant (Ka) through four-parameter logistic curve fitting (Figures 3A, B). ELISA results indicated that the Ka values of NC81 and NC89 were 3.54 × 10^−6^ M and 2.8 × 10^−6^ M, respectively. The diversity of CDR1 and CDR2 of Nbs has a certain effect on antigen-binding function (Muyldermans, 2013). However, CDR1 and CDR2 of Nbs were customized in this designed library and only randomized CDR3 to improve solubility expression, which may have an impact on antibody affinity. Thus, molecular docking was used to analyze whether the fixed CDR1 and CDR2 of Nbs exerted binding function (Lugano et al., 2018). As shown in Figure 3C, the results of FC showed that CD93 on HUVECs was more than twice that on 293T cells. In addition, the binding ability of NC81 and NC89 to HUVECs was examined with His antibody, and the results showed that NC81 and NC89 were able to bind specifically to HUVECs (Figure 3D).

### The thermal stability of anti-CD93 Nbs

The binding ability of Nbs treated at different temperatures to the antigen was examined by ELISA. As shown in Figures 3E, F, there was no significant change in NC89 and NC81 activity from 30°C to 80°C, and even at 80°C good binding ability was maintained, indicating that the Nbs have good thermal stability.

The Nbs frameworks in this study were derived from shark V-NAR, and normally shark-derived antibodies have high thermal stability. The structural changes of Nbs after different temperature treatments were investigated by circular dichroism (CD). The CD spectra of NC81 (Figure 3G) and NC89 (Figure 3H) treated at different temperatures had positive peaks at 192 nm and negative peaks at 203 nm. The positive peak at 192 nm represents the α-helix and the negative peak at 203 nm represents the β-fold. The results showed that the positive peak at 192 nm and the negative peak at 203 nm did not change significantly under 30°C–70°C treatment, indicating that Nbs still maintained their normal structure at 30°C–70°C. Meanwhile, NC81 showed a slight shift of the positive peak at 192 nm and the negative peak at 203 nm at 80°C, while NC89 maintained a similar structure. This suggests that NC89 may have higher thermal stability than NC81.

### Anti-CD93 Nbs inhibits HUVECs proliferation and migration

**FIGURE 4:**
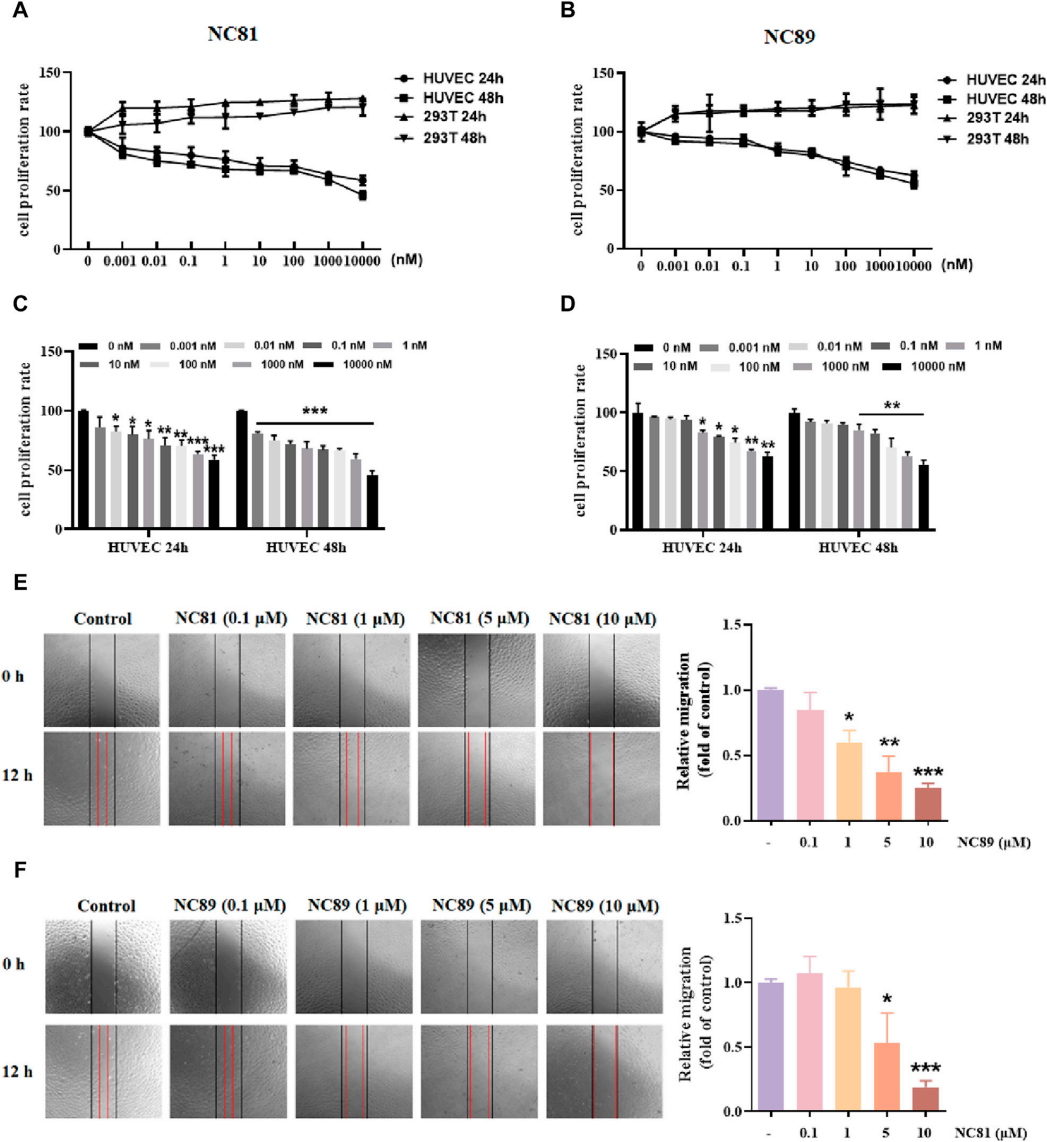
Anti-CD93 Nbs inhibit HUVECs proliferation and migration **(A)**. MTT assay. NC81 specifically inhibits HUVEC proliferation (n = 3). **(B)** NC89 specifically inhibits HUVEC proliferation (n = 3). **(C)** Quantitative analysis of NC81 inhibition of HUVEC proliferation (n = 3). **(D)** Quantitative analysis of NC89 inhibition of HUVEC proliferation (n = 3). **(E)** Migration assay. NC81 dose-dependently reduced the migration of HUVECs. Quantitative analysis on the right. (Original magnification × 100). **(F)** NC89 dose-dependently reduced the migration of HUVECs. Quantitative analysis on the right. (Original magnification × 100). Data are expressed as mean ± SEM. *p < 0.05, **p < 0.01, ***p < 0.001 *versus* Control.

As shown in Figures 4A–D, the MTT results showed that anti-CD93 Nbs NC81/NC89 significantly reduced the proliferation of HUVECs in a dose-dependent manner (*p* < 0.05). However, in 293T cells, there was no significant change in the results of the control and test groups (Figures 4A–D). The results showed that NC81 and NC89 could effectively and specifically inhibit the proliferation of HUVECs.

The effect of anti-CD93 Nbs NC81/NC89 on HUVEC migration was observed by scratch assay. As shown in Figures 4E,F, the gap width of Nbs treated group was more significantly reduced than that of the control group from 0 to 12 h (*p* < 0.05, *p* < 0.01, *p* < 0.001), indicating that NC81 and NC89 could dose-dependently inhibit the migration of endothelial cells. Furthermore, NC89 was able to significantly inhibit HUVEC migration at 1 μM.

### Tube formation evaluation

**FIGURE 5:**
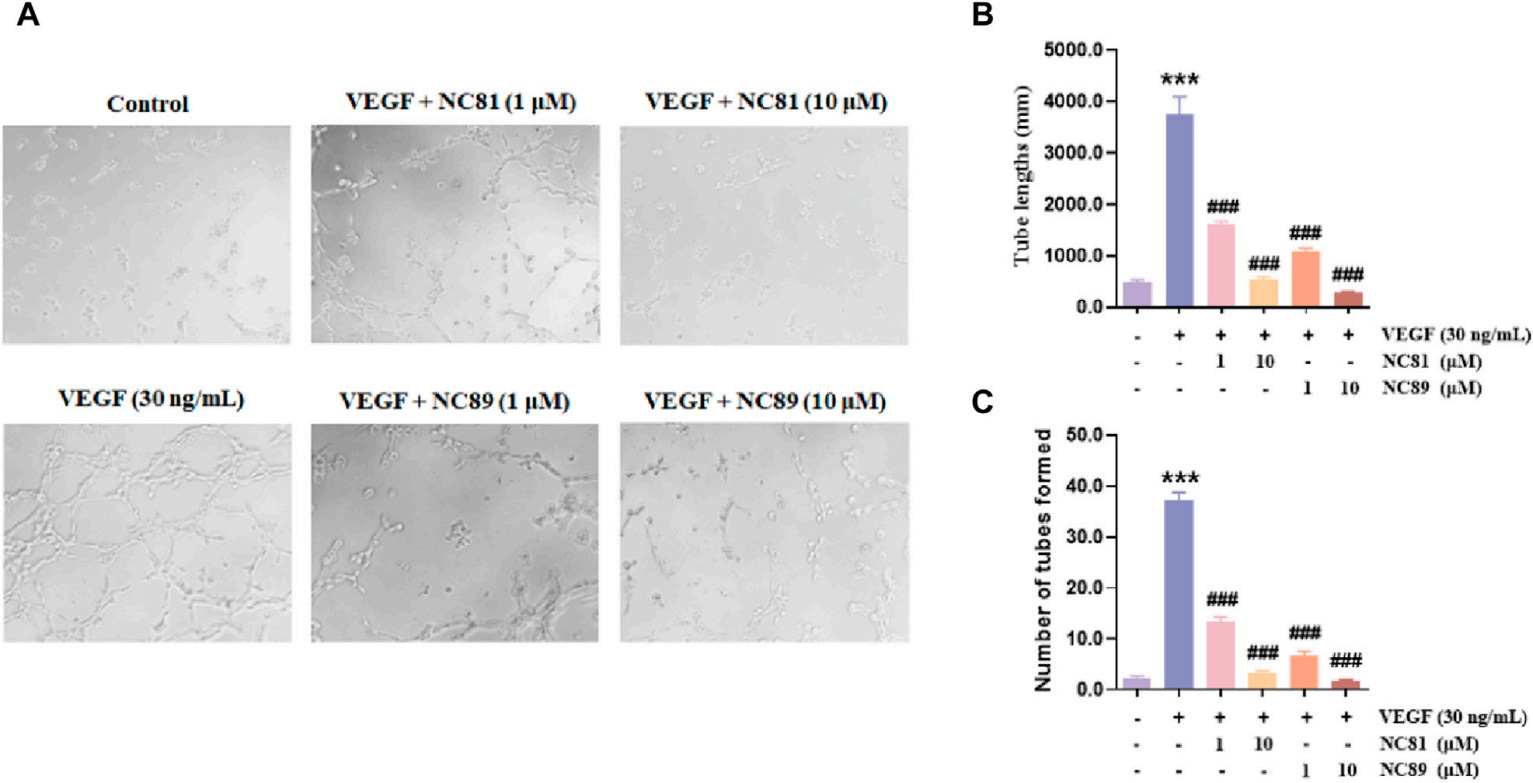
Inhibitory effects of the anti-CD93 Nbs on tube formation. **(A)** HUVECs were incubated with PBS and VEGF as negative and positive controls, respectively, and various concentrations (1 μM and 10 μM) of the Nbs were used (n = 3). (Original magnification × 100). **(B)** Quantitative analysis of tube length (n = 3). **(C)** Quantitative analysis of tubes formed (n = 3). Data are expressed as mean ± SEM. ***p < 0.001 *versus* Control; ^###^ *p* < 0.001 *versus* VEGF Group.

Although angiogenesis is a complex process in several kinds of cells, the maturation of endothelial cells into a capillary tube is a critical early step (Carmeliet, 2003; Lutsenko et al., 2003; Ferrara, 2010; Saharinen et al., 2011). To investigate whether the anti-CD93 Nbs NC81/NC89 could inhibit tube formation, HUVECs were cultured on Matrigel in the presence of different concentrations of NC81/NC89 (1 μM and 10 μM) and 30 ng/mL VEGF (Figures 5A–C). As shown in Figures 5A–C, there was obvious tube formation after the addition of VEGF, and capillaries were found to gradually disappear with increasing added NC81/NC89 concentration. Statistical results on tube lengths and numbers of nodes revealed that NC81 and NC89 significantly inhibited angiogenesis in HUVECs in a concentration-dependent manner (*p* < 0.001).

### Anti-CD93 Nbs increases vascular permeability

**FIGURE 6:**
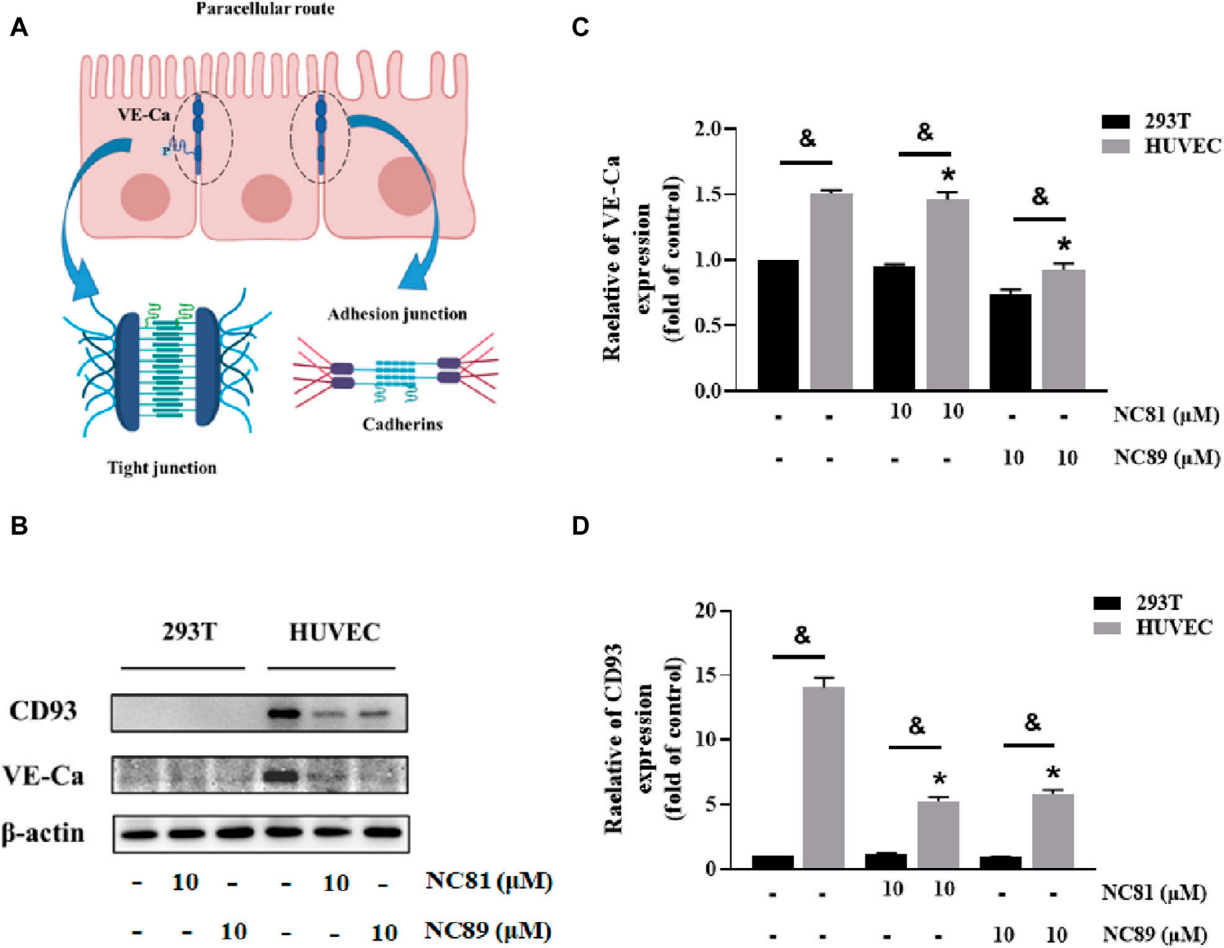
Anti-CD93 Nbs increase vascular permeability **(A)**. Schematic representation of intercellular tight junctions and adhesion junctions. **(B)** The expression of CD93, and VE-cadherin was detected by Western blot, and β-actin was used as control. The results represent at least three independent experiments. **(C)** Quantitative analysis of CD93 protein expression. The protein expression of CD93 was normalized to basal β-actin (n = 3). **(D)** Quantitative analysis of VE-cadherin protein expression. The protein expression of VE-Cadherin was normalized to basal β-actin (n = 3). Data are expressed as mean ± SEM. *p < 0.05 *versus* Control; ^&^ *p* < 0.05 *versus* 293T.

It has been shown that CD93 increases vascular permeability by inhibiting VE-Ca proteins and thereby increasing vascular permeability (Lugano et al., 2023) (Figure 6A). As shown in Figures 6B–D, the addition of 10 μM NC81 and NC89 to HUVECs significantly reduced the protein expression of CD93 and VE-Ca compared to the control, whereas no significant changes were observed in 293T cells.

### Docking results

**FIGURE 7:**
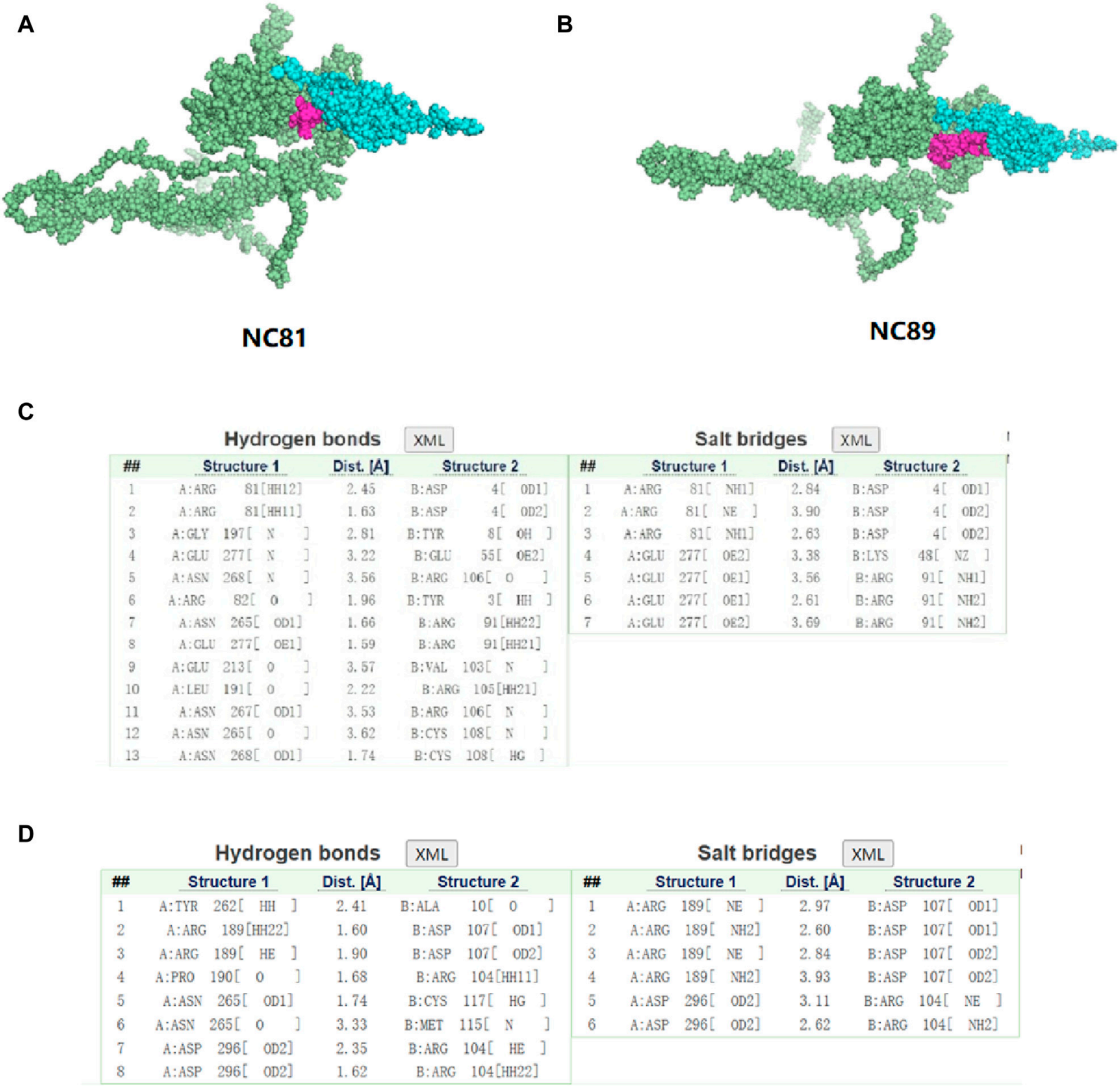
Three-dimensional structure simulation and docking analysis of anti-CD93 Nbs **(A)**. Three-dimensional structure of the CD93-NC81 complex. **(B)** Three-dimensional structure of the CD93-NC89 complex. **(C)** Interacting residues of the CD93-NC81 complex. **(D)** Interacting residues of the CD93-NC89 complex.

In order to study the interaction between NC81/NC89 and CD93, the NC81-CD93 and NC89-CD93 complexes were simulated with HADDOCK. HADDOCK, a semiflexible docking method, is suitable for docking construction of antigens and Nbs (Skottrup, 2017). The extracellular domain of CD93 was selected to dock with solvent-exposed residues of NC81/NC89, and the CD93-NC81/CD93-NC89 complex was modeled by HADDOCK. According to HADDOCK, its Z-score indicates how many standard deviations this structure is from the mean in terms of scores (the more negative the better). The evaluations retrieved from HADDOCK were −2.0 for the Z-score of CD93-NC81 with an RMSD of 0.5 ± 0.3 concerning the overall lowest energy structure, and −2.0 for the Z-score of CD93-NC89 with an RMSD of 0.7 ± 0.4 concerning the overall lowest energy structure, (Figures 7A,B). These results indicate that the complex structure model population obtained has high confidence. The optimal model cluster was assessed by PDBePISA to explore the interaction residues of NC81/NC89 and CD93 (Daniele et al., 2023). The paratopes on IC81 were made up of the following residues: ASP4, TYR8, GLU55, ARG106, TYR3, ARG91, VAL103, ARG106, CYS108 and LYS48. The epitopes on CD93 that bind to NC81 are ARG81, GLY197, GLU277, ASN268, ARG82, ASN265, GLU213, LEU191 and ASN267. The paratopes on NC89 were made up of the following residues: ALA10, ASP107, ARG104, CYS117, and MET115. The epitopes on CD93 that bind to NC89 are AYR262, ARG189, PRO190, ASN265 and ASP296. The interactions between CD93 and NC81/NC89 could be classified into hydrogen bonds and salt bridges. Interestingly, only ASN265 was bound to both NC81 and NC89 among the binding sites of CD93, and in the Nbs not only the CDR3 region but also some amino acids in the framework were involved in binding. (Figures 7C,D).

## Conclusion and discussion

Synthetic libraries are used for scientific research and antibody discovery with advantages in terms of short production cycles and low costs. To improve screening efficiency, many high-throughput screening techniques have been developed, such as yeast or bacterial two-hybrid techniques, and screening of mammalian cells or yeast surface display techniques by flow cytometry (Suzuki et al., 2018; Carrara et al., 2022). However, library construction using phage display techniques is still the dominant method for screening target antibodies (Yan et al., 2014; Ferrari et al., 2020). The library used in this study was a shark-derived phage display library, and we introduced diversity in the CDR3 region of the library by NNK. Nbs of >95% purity were obtained using Ni-column purification, with yields of 8.6 mg/L and 2.35 mg/L for NC81 and NC89, respectively. Compared with other studies, our method may be more practical in terms of protein yield and production cost. The binding affinity of the Nbs isolated from the immune library generally ranges from 10^6^ to 10^12^M^-1^ (Coppieters et al., 2006; Plagmann et al., 2009). CD93 binding and affinity assays indicate that NC81 and NC89 are within the range of affinity and have some binding capacity for CD93 protein. In addition, Nbs maintained their structural stability and their ability to bind antigens at high temperatures as measured by ELISA and CD spectroscopy. In future studies, mutation strategies for the CDR2 and CDR1 regions will be attempted to further improve the diversity of the library and the affinity of the Nbs.

We also investigated the potential anti-angiogenic activity *in vitro*. First, NC81 and NC89 were able to directly inhibit proliferation, migration, and tube formation on Matrigel by HUVECs. In addition, regulation of vascular permeability is essential for dynamic homeostasis between tissues and organs and facilitates increased transmission of macromolecular drugs as well as immune cells. It has been shown that knockout mice with CD93 have defective endothelial junctions and increased vascular permeability, that VE-Ca is an essential component of the tight junction (TJ) and adhesion junction (AJ), and that CD93 maintains endothelial cell stability by inhibiting phosphorylation and internalization of VE-Ca. The molecular mechanism by which NC81 and NC89 regulate vascular permeability was verified by WB, which showed that NC81 and NC89 blocked the expression of CD93 and VE-Ca, thereby increasing vascular permeability. These results suggest that NC81 and NC89 could be used as promising anti-angiogenic as well as vascular permeability increases. Because of the many advantages of the Nbs, we are therefore more interested in comparing the antitumor activity of different CD93 Nbs *in vivo*. Further studies are in progress in this regard.

To explore the sites of NC81/NC89 and CD93 binding, we performed protein-protein docking (Genst et al., 2004). The docking results show that NC81 binds to CD93 mainly through hydrogen bonds and salt bridges formed by the CDR1 and CDR2 regions and that only residues ARG 91, VAL 103, and ARG 106 in CDR3 interact with the active site of CD93; thus, the CDR1 and CDR2 regions contribute to the formation of a complex between NC81 and CD93. In contrast, NC89 binds to CD93 mainly through residues ASP107, ARG104, and MET115 in CDR3, and the CDR1 and CDR2 regions are not involved in its binding to CD93.

## Data Availability

The original contributions presented in the study are included in the article/Supplementary material, further inquiries can be directed to the corresponding author.

## Abbreviations

CD93, Cluster of Differentiation 93; Nbs, nanobodies; ELIS, Aenzyme-linked immunosorbent assay; FC, Flow cytometry; HUVEC, shuman umbilical vein endothelial cells; WB, Western Blot; VE-Ca, VE-cadherin; mAbs, monoclonal antibodies; FBS, Fetal bovine serum; HEK293, human embryonic kidney cells; ANOVA, analysis of variance; LSDl, east significant difference; PCR, polymerase chain reaction; Ka, association constant; CD, circular dichroism; TJ, Tight junction; AJ, Adhesion junction.
